# Performance of Cutting Tool with Cross-Chevron Surface Texture Filled with Green Synthesized Aluminium Oxide Nanoparticles

**DOI:** 10.1038/s41598-019-54346-0

**Published:** 2019-11-28

**Authors:** Sathiya Narayanan N., Baskar N., Vedha Hari B. N., Rohith Sankaran, Ramya Devi D.

**Affiliations:** 1 Department of Mechanical Engineering, Saranathan College of Engineering, Trichy, 620012 Tamil Nadu India; 2 Pharmaceutical Technology Laboratory # 214, Anusandhan Kendra-II, School of Chemical & Biotechnology, SASTRA Deemed University, Thanjavur, 613401 Tamil Nadu India; 3 School of Mechanical Engineering, SASTRA Deemed University, Tanjavur, 613401 Tamil Nadu India

**Keywords:** Mechanical engineering, Other nanotechnology, Mechanical engineering, Other nanotechnology

## Abstract

The newer methodology to improve the performance of cutting tool is by the constructive method of micro-texturing and green synthesized nanoparticles into the texture gaps for self-lubrication. Cross-chevron textures were made on the rake face of the cemented carbide tool using Neodymium Doped Yttrium Aluminium Garne (Nd-YAG) laser texturing machine. The environmentally friendly, non-hazardous and rapid method of producing nanoparticles was followed to produce Al_2_O_3_ nanoparticles. Various techniques used for characterizing the synthesized Al_2_O_3_ nanoparticles are Potential of Hydrogen (pH), Fourier Transform Infra-Red Spectroscopy Analysis (FTIR), Dynamic light scattering (DLS) and X-Ray Diffraction (XRD). The XRD shows the presence of required functional groups and the size of nanoparticles in the range of 500–550 nm. This article discusses the effect of textures, with and without nanoparticles filled on the texture gaps of the cemented carbide tool on the main cutting force, thrust force and co-efficient of friction while machining austenitic stainless steel 304. The combined effect of surface texturing and lubrication of Al_2_O_3_ nanoparticles enhanced the performance of the cutting tool compared with the conventional and textured tool.

## Introduction

Austenitic stainless steel is always considered as a difficult to machine material due to its properties like work hardening and low thermal conductivity^1^. It carries away a part of the tool when subjected to machining due to its strong bonding nature with the tool material. Even though austenitic stainless steel has moderate machinability, its usage is enormous worldwide. Parts requiring high corrosion resistance combined with high strength are manufactured using austenitic stainless steel. Austenitic stainless steels are commonly preferred for producing tanks, vessels, pipes, pharmacy equipment and automobile parts^2,3^. The green synthesized nanoparticles filled in the texture gaps of the cutting tool would replace the need for cutting fluids during the machining operation.

Usage of proper lubricants can reduce the tool wear while machining austenitic stainless steel. Green cutting fluids combined with conventional cutting fluids prove to be of great value and have shown excellent results in reducing tool wear^4^. Proper online monitoring systems using acoustic emissions to study the effects of work hardening were developed. Optimized speed conditions are conducive in reducing the tool wear, thereby increasing the tool life and the quality of the product^5^.

Newer methods are always in the study to reduce tool wear. One of the primary ways is surface coating. Coatings reduce the cutting pressure and reduce the friction between the tool and the workpiece. The optimum composition of nanocoating significantly improves the machining performance when applied on tungsten-free carbide tool^6^. The implementation of coatings shrinks the contact lengths between the tool and the workpiece and hence lessens the compression stress and cutting forces^7^. The more recent development in the area of coatings is nanocoating. TiAlN/CrN nanocoating with different periodicities applied on the tool were carried out, and among them, 2 mm periodicity showed strong coating characteristics and surface morphology^8^. A study about wear and diffusion process in the multilayer coating of Ti-TiN-(Ti, Al, Si)N composite was carried out which resulted in the formation of wear-resistant nano- and sub nanolayers of (Ti, Al, Si)N thus resulting in better cutting properties of the carbide tool when compared to the conventional TiN coatings^9^.

Another method to reduce the tool wear and thereby improve the performance is surface texturing. Surface texturing is a typical self-lubricating technique by creating different patterns on the rake and flank faces of the cutting tools. Surface texturing is explained in two parts: (i) various sources for the creation of surface textures and (ii) types of textures reported. Metal forming, micro/nano machining, electrochemical machining, mechanical micromachining and thermal energy micromachining are the various sources for creating surface textures as discussed in the literature^10^. From the list of available sources, electro discharge machining (EDM) and laser surface texturing under thermal energy micromachining are the commonly used techniques due to their easy availability, accuracy and lesser time for creating the textures. Laser surface texturing is competent in creating any kind of textures irrespective of the complexity of the texture pattern. Different texture patterns like parallel and perpendicular textures, micro holes in the form of array textures, crossline textures, dimple and channel textures, hybrid textures, areal and linear textures, elliptical grooves, circular forms, rectangular forms and chevron textures created on the flank face, rake face, and flank and rake face at micro as well as nano level depths are commonly reported^11^.

Further, the textured gaps were filled with solid lubricants MoS_2_ and CAF_2_ to improve the performance and reduce the wear. The perpendicular pattern on the tool gave good results in terms of reduced cutting forces and tool wear when compared to the parallel and plain non-textured tool during the machining of Ti-6Al-4V. The results were compared experimentally and with the help of simulation results^12^. The more recent development is the chevron pattern which gave better results than the perpendicular pattern when inscribed on carbide tool during the machining of Ti-6Al-4V. In addition, the coating was done, and the tool with a combination of coating and chevron pattern gave the best result in terms of cutting force and tool wear^13^. Apart from patterns, micro holes on tools of different sizes also prove to reduce the machining forces and the degradation of the tool significantly^14^. The pitch distance was the most dominant parameter for the reduction of cutting forces; better lubricity was observed due to the effectiveness of the circular pits between the pitch distances^15^. The cutting tool performance improved due to the reduction of actual chip-tool interface contact length and the presence of lubrication file in the interface^16^.

The micro elliptical texture was made on the rake and flank faces of the cemented carbide cutting tool for the dry machining of Ti-6Al-4V material, and the texture gaps were filled with molybdenum disulphide. Experimental results showed a reduction in the cutting forces, working temperatures, chip thickness ratio and the tool life improvement of 10–15% and 10–30% as reported in the literature^17^. Chen ZM *et al*.^18^ discussed the derivative cutting and nonderivative cutting textures and simulated the effect of various parameters of micro-textures for the orthogonal cutting of Ti-6Al-4V. AdvantEdge simulation software was used for finding out the influence of groove width and texture rate and reported that the low texture rate showed a reduction in terms of cutting force and temperature, high texture rate and size that has adverse effects. Tungsten disulphide (WS_2_) nano-textured and soft-coated self-lubricating tools were proposed in the literature^19^. Authors reported the formation of lubrication film at the tool-chip interface. Due to the smearing action of nano-texture, the cutting forces and temperatures were reduced. A less frictional force was observed in nano-textured tools due to reduced contact length. The tool life was prolonged with the help of anti-adhesive effects shown by the tungsten disulphide nano-textured and soft-coated self-lubricating tool compared with the conventional and nano-textured tools for machining high hardened steel materials. Optimum width to depth ratio, edge distance and width of the microgrooves were reported using computational simulation using AdvantEdge software. The performance of the micro-textured tool showed lesser values of cutting forces, temperature, frictional behaviour of chip-tool interface and energy consumption for machining Ti-6Al-4V with micro-textured cemented carbide tool as detailed in the literature^20^.

The concave surface of the groove textures makes the storage space of the reservoir and prevents the outflow of lubricating oil and chips^21^. Material adhesion and friction coefficient get reduced in textured tool due to the obstruction of the chip flow compared to the non-textured tool^22^. Usage of nanoparticles as adequate lubrication for machinery and tooling has been in the study for a while. The study of aluminium oxide nanoparticles suspended in SAE 20 W40 oil concluded that the addition of nanoparticles reduced friction by reducing the contact area, leading to wear tracks^23^. The study of aluminium oxide nanoparticles in SAE EP 90 oil for heavy machinery was carried out. A thorough investigation of the rheological properties of the oil was carried out and it was concluded that the addition of Al_2_O_3_ nanoparticles improved the rheological properties^24^. Chemical methods are used for producing nanoparticles^25,26^, but with the harmful effects and cost involved, the focus is shifting toward green synthesis. Green synthesis is one of the alternate ways to prepare nanoparticles. Green synthesis is a less harmful and cost-effective process compared to chemical methods^27^.

A comprehensive study of the mechanism related to the synthesis of nanoparticles from plants which is bio reduction and the factors that affect their synthesis and needs in various industries was highlighted along with the environmental effects and significant cost reduction compared to chemical methods. These studies proved that the green synthesis of nanoparticles is a quick, easy, cost-effective and less harmful method^28^. Various types of metal oxide nanoparticles were produced from many plants. Neem leaves have been one of the go-to medicinal plants in the Indian subcontinent. They can also be used for the generation of a wide range of metal nanoparticles by adding salts of the required metal, and the process of bio reduction of phytochemicals present in plants gives out the metal ions. The synthesis of bimetallic gold cord silver nanoparticles from neem leaves was studied. The mechanism of bio reductions of phytochemicals like flavanones and terpenoids to generate the metal nanoparticles was shown in detail, and the research opened up ways to optimize the synthesis to get better yields. The primary characterization test (XRD, FTIR and Scanning Electron Microscope (SEM)) to ensure the stability of aluminium oxide nanoparticles generated from rose leaves showed that nanoparticles are more stable with a low toxicity level compared to its chemical counterpart^29^.

This research mainly focuses on the lubrication properties of aluminium oxide nanoparticles synthesized using neem leaves. Four different tools were used, tool number 1 is plain tool, tool number 2 without texturing and with Al_2_O_3_ nanoparticles coated in the rack face, tool number 3 is subjected to a laser texturing process with the novel cross-chevron pattern inscribed on the rake face and not filled with nanoparticles and finally, tool number 4 is the combination of both cross-chevron texturing and Al_2_O_3_ nanoparticles filled in the texture gaps. Performance of these four different tribological tools was compared with the main cutting force, thrust force and coefficient of friction.

## Materials, Methods and Machining

The materials used in the research, material purchase details, laser machine setup details, surface texture dimensions, methodology incorporated for the formation of the textured tool, green synthesis of nanoparticles and semiautomatic lathe machine details are discussed.

### Materials

Austenitic stainless-steel grade 304 (length 150 mm, Ø 30 mm diameter rods are procured from Pandi Steels Pvt Ltd, Madurai, India) was considered as workpiece material. The physical composition is mentioned in Table 1. Cemented carbide tool (K-Grade CNMA 120404, KORLOY made, GTK Tools, Chennai, India) was chosen as tool material. CNMA 120404 – ‘C’ shape with an included angle of 80°; ‘N’-turning insert with 0° clearance angle; ‘M’-tolerances; ‘A’-turning insert with cylindrical hole; cutting edge length – 12 mm; thickness – 4 mm; nose radius – 0.4 mm. Neem leaves collected in the campus, distilled water generated in-house, aluminium chloride, ethanol solution, alumina reagent and methyl orange indicator (procured from Himedia Laboratories Pvt Ltd) were used in the process of producing Al_2_O_3_ nanoparticles.

**Table 1:** Physical composition of austenitic stainless-steel grade 304^30^.

Element	Cr	Ni	C	Si	Mn	Mo	Fe
Wt (%)	18.577	8.29	0.029	0.273	2.25	0.223	Balance

### Methods

#### Surface texturing

Nd-YAG laser (Lee laser, TQ9005) was used to fabricate surface texturing on the PCD tool. Laser machine parameters are as follows: speed of scanning V = 40 mm/s, energy E = 30 mJ, laser frequency f_L_ = 25 kHz and duration for the pulses t = 25 ns (optimized laser texturing machine parameters). Cross-chevron texture parameters are of depth −200 µm, texture included angle −40°, vertical distance −1.5 mm and horizontal distance −1.5 mm. The X and Y direction of the laser source was controlled using controllers and integrated with a personal computer to ensure that the correct path is followed as per the texture pattern.

#### Synthesis of Al_2_O_3_ nanoparticles

**Figure 1:**
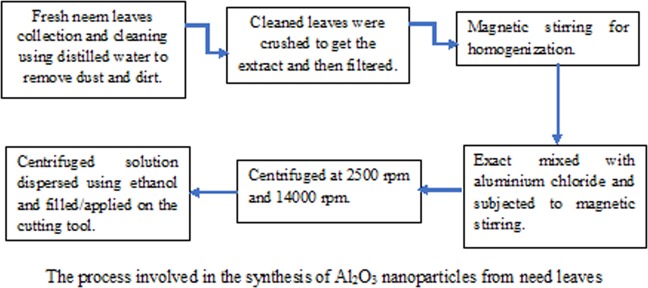
The Process involved in the synthesis of Al_2_O_3_ nanoparticles from neem leaves. Fresh neem leaves were collected and washed twice using disposable and distilled water, followed with crushing the leaves. The extract was homogenized to achieve uniform mixing and then mixed with saturated aluminium chloride. The change in color ensures the photochemical reaction, which results in nanoparticles. The mixture was centrifuged at low and higher speeds and then dispersed using ethanol.

Fresh neem leaves were plucked and thoroughly washed twice using disposable water and then with distilled water to remove dirt and debris. The washed leaves were crushed using a mortar and pestle to get the extract, which then was filtered using a muslin cloth. The filtered extract was homogenized using magnetic stirrer (2 ml, REMI, Laboratory Instruments, Mumbai, India) for 20 minutes to achieve uniform mixing. This extract was then mixed with a saturated solution of aluminium chloride drop by drop and again subjected to magnetic stirring for 2 hours. The pH change after stirring indicates that a reaction between phytochemicals and metal salts has taken place, resulting in the release of nanoparticles ions. This mixture was then centrifuged at low speed (2500 rpm) for 20 minutes (REMI R-8C Laboratory Centrifuge) and then at high speed (14000 rpm) for 30 minutes (REMI C-24 Plus). The centrifuged solution was then dispersed using ethanol and kept for filling into the texture gaps (Fig. 1). The synthesized Al_2_O_3_ nanoparticles were filled into the texture gaps using a syringe. Uniform spreading into the texture gap was ensured with the aid of the syringe needle. A mild temperature of 30 °C was maintained, after which ethanol evaporated slowly leaving out the Al_2_O_3_ nanoparticles alone into the texture gap. The plain tool was coated with Al_2_O_3_ nanoparticles using a similar procedure.

### Instrumental analysis

The measurement of the particle size of the nanoparticles was obtained by electroacoustic dynamic light scattering principle (Malvern Nano series ZS, UK). The FTIR analysis was performed in by ATR mode for measuring the vibrational spectroscopy of the nanoparticles, in the wavelength range of 400–4000 cm^−1^ at room temperature (Spectrum 100, Perkin Elmer, USA). The angles and intensity of the diffracted rays (XRD) of the nanoparticles as a specimen are measured with an applied current of 20 mA and voltage of 30 kV, sweep of 10–80° at an angle 2θ (D8 Focus, Bruker AXS, Germany). The synthesized nanoparticles were dried on a glass slide and subjected to SEM (JSM 6701 F, JEOL, Japan) analysis to check for its particle size and shape.

### Machining

**Figure 2:**
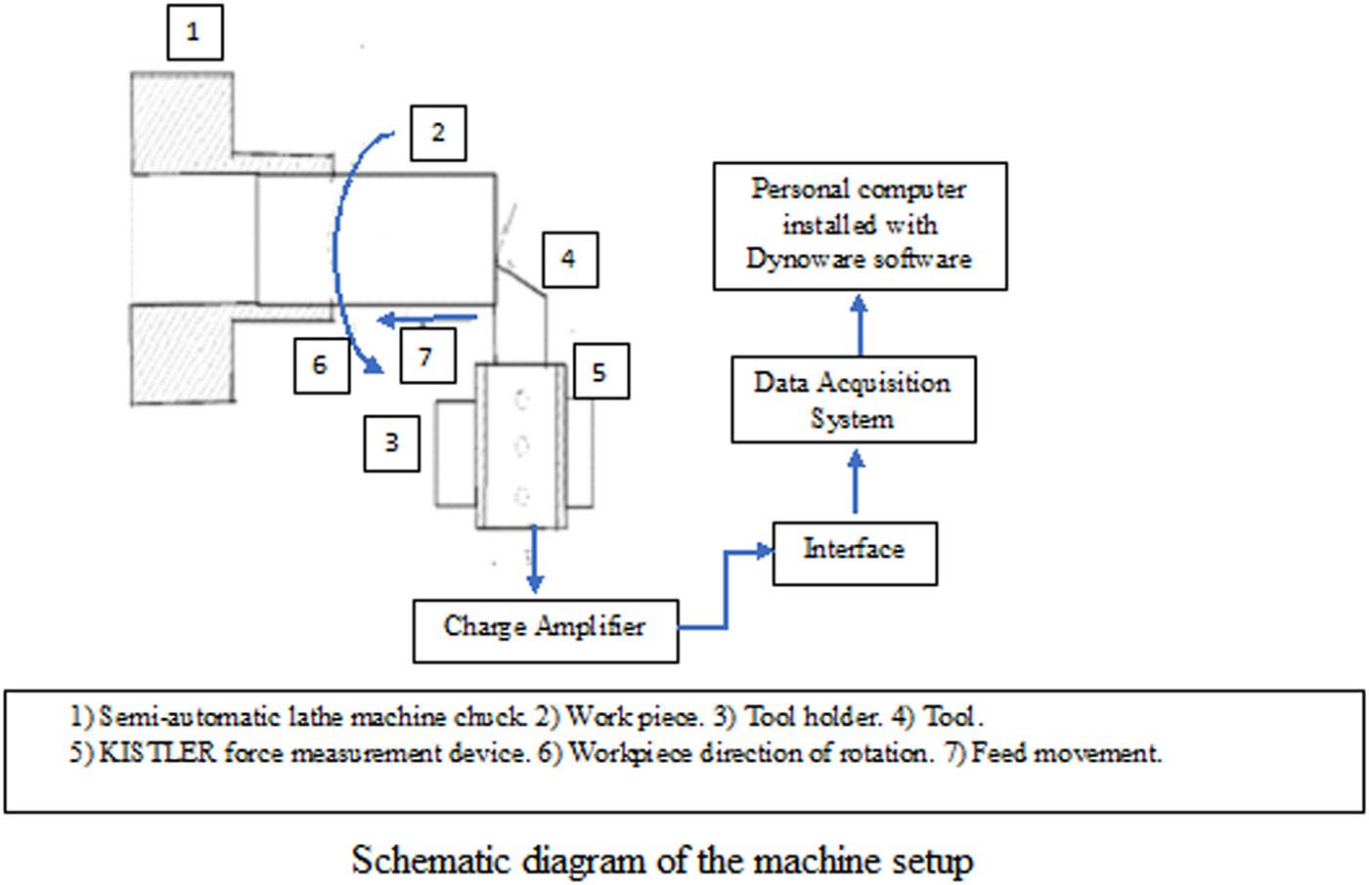
Schematic diagram of the machine setup. Schematic diagram of the semi-automatic lathe machine attached with 3-Component force measurement setup for measuring the cutting forces.

The turning was carried out on PINACHO Semi-Automatic lathe attached to a Kistler-3 component, and piezoelectric dynamometer (carried out at PSG College of Technology, Coimbatore, India, schematic diagram is shown in Fig. 2) was used to measure the cutting forces. The machining parameters and experimental conditions are described in Table 2. Machining was carried out with four different types of tools, namely plain, non-textured with Al_2_O_3_ nanoparticles coating, plain textured and textured with Al_2_O_3_ nanoparticles filling. The forces were measured thrice to ensure accuracy and repeatability for each case.

**Table 2:** Experimental conditions.

No. of experiments	36 Experiments (*n* = 3)
Type of tools used	Plain tool (PT), non-textured surface filled with Al_2_O_3_ nanoparticles (NTN), textured tool (TT), textured and filled with Al_2_O_3_ nanoparticles (TN)
Coolant	Dry machining
Cutting velocity – *V*_c_ (m/min)	60, 80, 100
Feed rate – *f* (mm/rev)	0.25
Depth of cut – *a*_p_ (mm)	0.5

## Results and Discussions

### Characterization of Al_2_O_3_ nanoparticles

#### Potential of hydrogen (pH) analysis

The potential of hydrogen (pH) for the plant extract and the prepared nanoparticles was analyzed separately using pH meter. The initial pH of the plant extract was found to be 6.4. After the addition of 5 ml of the extract to 15 ml of saturated aluminium chloride solution, followed by 2 hours of stirring, the pH reduced to 3.0. The change in pH reveals the reaction between the phytochemicals of plant extracts and the metal salt to convert the metal oxide nanoparticles.

#### Test for aluminium and hydroxide group

The green synthesized nanoparticles were subjected for qualitative analysis to confirm the formation of aluminium oxide nanoparticles through a green synthesis process. The nanoparticles are mixed with alumina reagent and methyl orange indicator separately to confirm the presence of aluminium and hydroxide groups. The formation of yellow precipitate indicated the presence of aluminium whereas the formation of the red lake indicated the presence of the hydroxide group and confirmed the formation of Al_2_O_3_ nanoparticles.

#### Fourier transform infrared spectroscopy (FTIR) analysis

**Figure 3:**
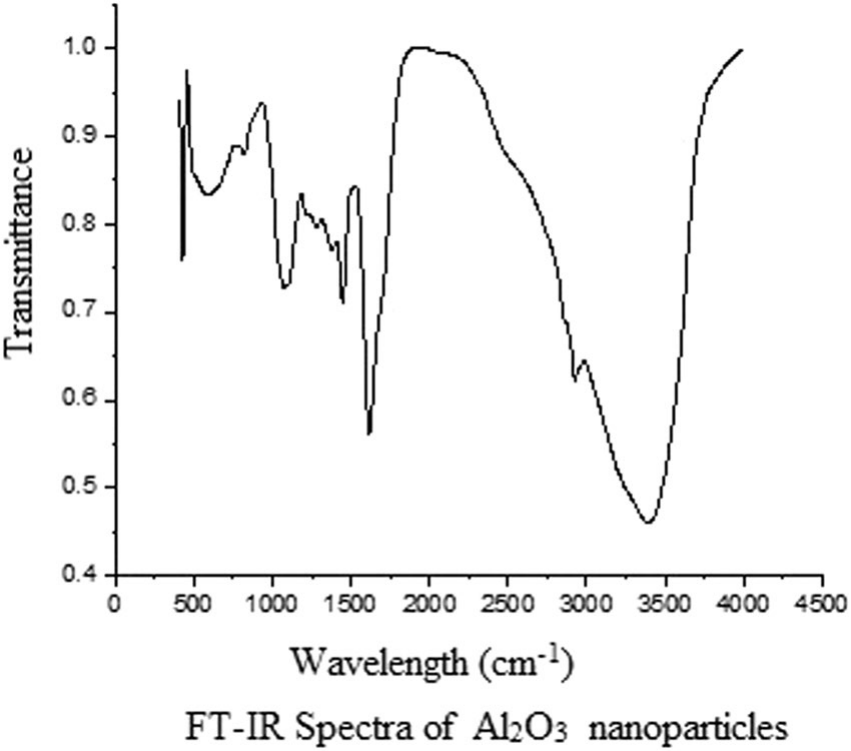
FT-IR spectra of Al_2_O_3_ nanoparticles. Fourier transforms infrared spectroscopy analysis was carried out. The highest peak obtained at 3600 cm^−1^ is related to O-H stretching; the next, highest peak occurs at 1700–1800 cm^−1^ are responsible for C=O stretching, 600–700 cm^−1^ confirms the Al-O-Al bond at the gamma phase of alumina.

FTIR measurement was obtained to find the possible incumbent biomolecules for the formation of metal (Al) nanoparticles. Figure 3 shows the FTIR spectrum of Al_2_O_3_ nanoparticles; the highest peak obtained at 3600 cm^−1^ is related to O-H stretching; the second highest peak occurring at 1700–1800 cm^−1^ is responsible for C=O stretching. Peak wavelength around 600–700 cm^−1^ confirms the Al-O-Al bond at the gamma phase of alumina. Thus, Al_2_O_3_ phase contains the tetrahedral as well as the octahedral coordination.

#### Particle size and SEM analysis

**Figure 4:**
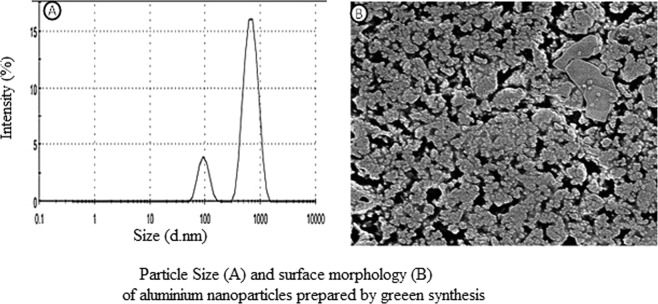
Size of the Al_2_O_3_ nanoparticles. (**A**) The green synthesized aluminium oxide nanoparticle subjected for particle size analysis by dynamic light scattering principle and showed an average size of 525.25 nm with a polydispersity index (PdI) of 0.547. (**B)** Surface morphology of prepared nanoparticle showed the particles are in a spherical shape and appears as clumps.

The green synthesized aluminium oxide nanoparticle subjected for particle size analysis by dynamic light scattering principle and showed an average size of 525.25 nm with a polydispersity index (PdI) of 0.547 as refereed in Fig. 4A. The particle distribution is found to be polydispersity as the PDI values > 0.5. This is also confirmed by Scanning electron microscopy analysis (Fig. 4B) where the surface morphology of prepared nanoparticle showed the particles are in a spherical shape and appears as clumps.

#### XRD analysis

**Figure 5:**
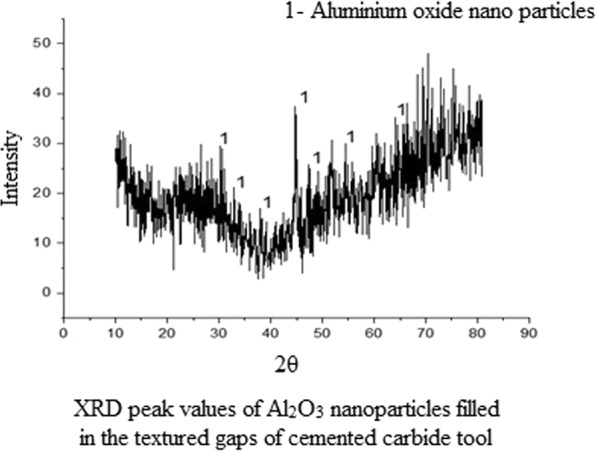
XRD peak values of Al_2_O_3_ nanoparticles filled in the textured gaps of cemented carbide tool. XRD analysis was carried out after filling the nanoparticles into the textured gaps of cemented carbide tool. The higher peaks in the graphs confirm the uniform filling of nanoparticles, the lesser peak height relates to other particles present in the tool.

The XRD analysis was conducted to interpret the structure of the synthesized aluminium oxide nanoparticles and was found to be α-aluminium oxide with a trigonal lattice structure formation. The peak values were obtained at the following intensities: 25.63°, 35.14°, 37.76°, 43.45°, 52.53°, 66.50°, 68.19°, as shown in Fig. 5.

### Surface morphology

**Figure 6:**
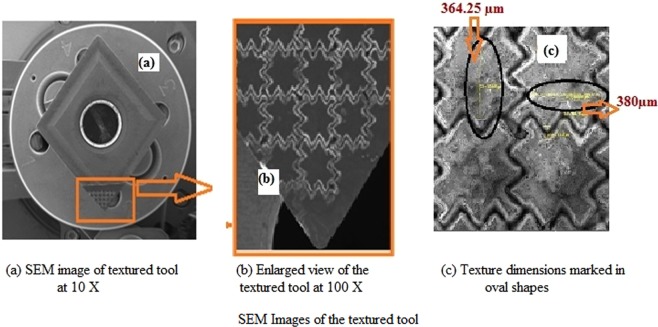
SEM Images of textured tool. (**a**) SEM image of texture made in the rake face of the cemented carbide tool. (**b**) Enlarged view of the textured tool. (**c**) Markings are made to show the vertical and horizontal distance between the consecutive textures (364.25 µm and 380 µm respectively).

**Figure 7:**
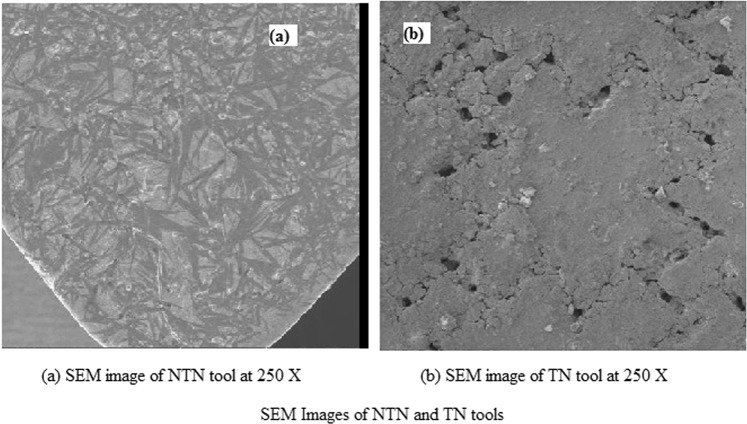
SEM Images of NTN and TN tools. (**a**) SEM Image of the non-textured cemented carbide tool applied with the nanoparticles over the rake face. (**b**) SEM Image of the cross-chevron textured tool filled with the nanoparticles in the gaps of the textures.

The necessary tribological property enhancement was reported^9–16^, by forming textures on the rake and flank faces on the tool. The adhesive wears due to the welding action of the workpiece and tool while performing at a higher temperature. Diffusion wear due to the dispersion and solubility nature of the tool and coolant which were referred to be debris entrapment would be reduced by the formation of the cross-chevron pattern and filling the textures with Al_2_O_3_ nanoparticles. Figure 6(a) shows the SEM image of the cross-chevron textured tool, (b) shows the enlarged image of the texture and (c) shows the vertical and horizontal dimensions which are marked in oval shapes with values of 364.25 µm and 380 µm, respectively. Thin-film lubrication that helps in reducing friction and tool wear was reported^10^ The non-textured tool surface applied with Al_2_O_3_ nanoparticles and the textured tool filled with Al_2_O_3_ nanoparticles in the texture gaps is shown in Fig. 7(a,b).

### Turning experiments

#### Cutting forces and average co-efficient of friction

**Figure 8:**
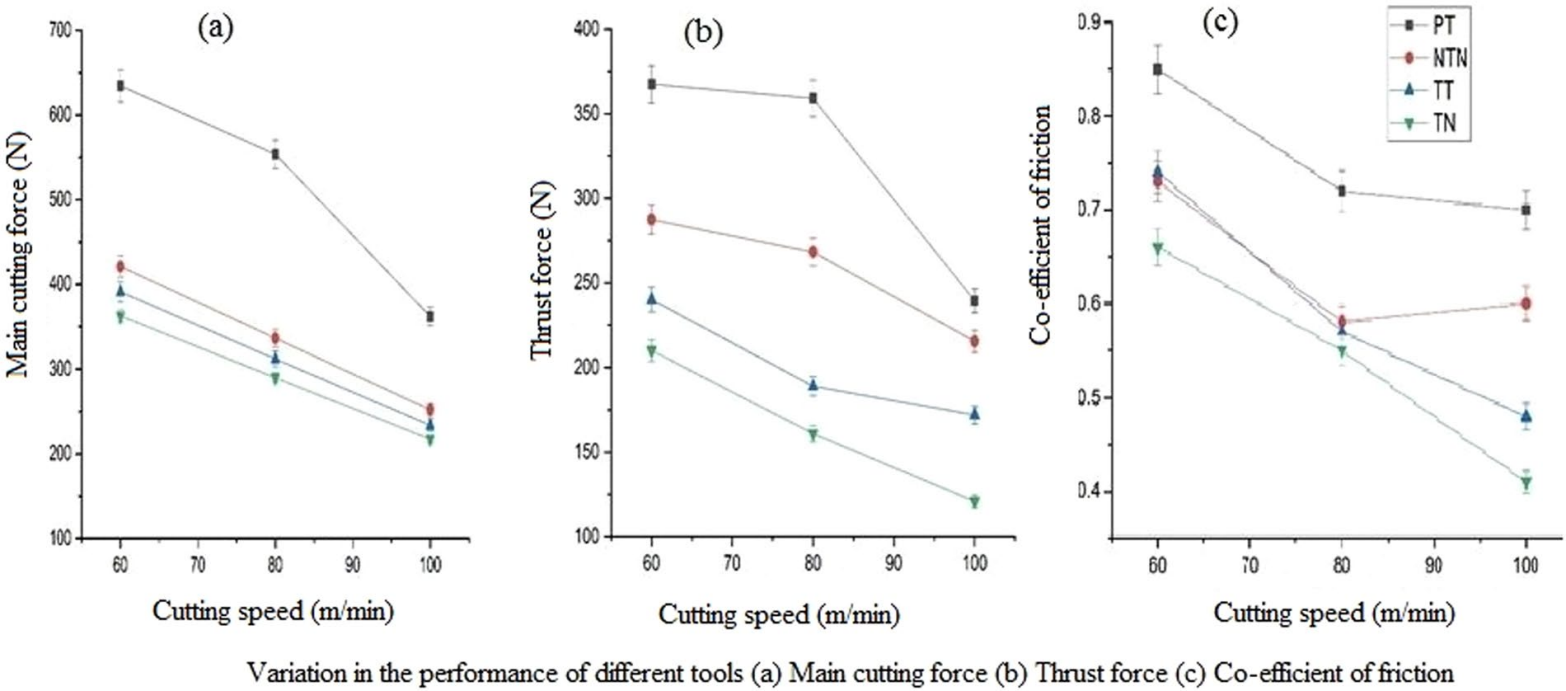
Variation in the performance of different tools. (**a**) The performance of the different tools at various cutting speeds concerning the main cutting force was shown. TN tool outperformed all other tools for machining with same conditions. (**b**) The thrust force obtained using different tools at various cutting speeds are shown. From the graph, it is evident that the TN tool required lesser thrust force compared with other tools. (**c**) Co-efficient of friction calculated is shown in graphical format. From the graph, it is evident that the co-efficient of friction values are lesser for TN tool compared with other considered tools.

The main cutting forces (F_z_) for the plain tool, non-textured tool surface applied with Al_2_O_3_ nanoparticles, cross-chevron textured tool and textured tool filled with Al_2_O_3_ nanoparticles are compared (see Fig. 8a). From the experimental results, it was observed that when the cutting speed increased between 60 m/min and 100 m/min, the main cutting forces showed reducing inclinations due to thermal softening of all the four tools being considered here. The non-textured tool surface applied with Al_2_O_3_ nanoparticles obtained higher main cutting forces than the textured tool and lesser than the plain tool. The nanoparticles were applied only over the rack face of the tool which was not enough to reduce the cutting forces. Lesser reduction in the percentage of forces was obtained in the textured tool because the textures near the cutting edge played a vital role by the hydrodynamic lift induced by the air gaps in the texture, and the textures far away from the cutting edge did not play its part. In the textured tool filled with Al_2_O_3_ nanoparticles, the nanoparticles had sufficient area to get filled in the texture gaps that helps in reducing the cutting forces compared with all other tools due to the combined effects of hydrodynamic lift and lubrication behaviour of Al_2_O_3_ nanoparticles filled in the textures.

For different cutting speeds, thrust force (F_y_) was related to the plain tool, non-textured tool surface applied with Al_2_O_3_ nanoparticles, cross-chevron textured tool and textured tool filled with Al_2_O_3_ nanoparticles (see Fig. 8b). It was observed that all the four tools followed the decremental trend like the main cutting force. During machining performed at a cutting speed of 80 m/min, the NTN, TT and TN tools showed higher percentage reduction of thrust forces, namely 25.32%, 47.37% and 55.17%, respectively, compared with machining carried out at cutting speeds of 60 m/min and 100 m/min. The surface texture and self-lubrication mechanisms of the tool worked well at medium cutting speeds rather than lower- and higher-level cutting speeds in this used combination of tool, workpiece and nanoparticle materials.

The average friction coefficient values were calculated by using the formula mentioned below [Zhang, 1988] for the plain tool, non-textured tool surface applied with Al_2_O_3_ nanoparticles, cross-chevron textured tool and textured tool filled with Al_2_O_3_ nanoparticles (See Fig. 8c).

$$\mu =\,\tan ({\rm{\beta}})=tan(\gamma +arctan(\frac{{F}_{y}}{{F}_{z}})$$

where μ = average friction coefficient, ${\rm{\beta}}$ = angle of friction, *γ* = rake angle of the cutting tool, *F*_*z*_ = main cutting force (N) and *Fy* =  thrust force (N).

## Conclusion

This article presents the co-effect of textures and Al_2_O_3_ nanoparticles on the cemented carbide tool during dry turning of austenitic SS304 materials. The Al_2_O_3_ nanoparticles are prepared by green synthesis, which is proved to be a cost-effective process. The characterization test, particle size analyzer, SEM and XRD ensure the presence of Al_2_O_3_ nanoparticles and confirm its size in nanoscales. The cutting forces and chip-tool interface friction coefficients are reduced by creating unique cross-chevron textures on the surface of the tool near the cutting edge. The textured tool (TT) prevents the formation of continuous chips and get deposited on the rake face of the tool, which slows down the adhesion area formation. In the case of TN tool, the combined effect of texturing and lubrication of Al_2_O_3_ nanoparticles enhances the performances in all aspects of machining. This research opens newer dimensions in coating and texturing of cutting tool and making of metal nanoparticles via natural synthesis to act as self-lubricants in various dry machining processes. The usage of green synthesized nanoparticles is non-hazardous, non-toxic and non-allergic and less cost-oriented, fulfil the laws and regulations and helps in providing a better working environment in production industries.

## Data Availability

The datasets generated during and/or analyzed during the current study are available from the corresponding author on reasonable request.

## References

[1] Endrino J, Fox-Rabinovich G, Gey C (2006). Hard AlTiN, AlCrN PVD coatings for machining of austenitic stainless steel. Surf. Coat. Technol..

[2] Kaladhar M, Subbaiah K, Rao C (2012). Machining of austenitic stainless steels - a review. IJMMM..

[3] Akasawa T, Sakurai H, Nakamura M, Tanaka T, Takano K (2003). Effects of free-cutting additives on the machinability of austenitic stainless steels. J. Mater. Process. Technol..

[4] Korkut I, Kasap M, Ciftci I, Seker U (2004). Determination of optimum cutting parameters during machining of AISI 304 austenitic stainless steel. Mater. Des..

[5] Sullivan DO, Cotterell M (2002). Machinability of austenitic stainless steel SS303. J. Mater. Process. Technol..

[6] Vereschaka AA, Vereschaka AS, Batako AD (2016). Development and research of nanostructured multilayer composite coatings for tungsten-free carbides with extended area of technological applications. Int J Adv Manuf Technol..

[7] Vereschaka AA, Bublikov JI, Sitnikov NN (2018). Influence of nanolayer thickness on the performance properties of multilayer composite nano-structured modified coatings for metal-cutting tools. Int J Adv Manuf Technol..

[8] Delisle DA, Krzanowski JE (2012). Surface morphology and texture of TiAlN/CrN multilayer coatings. Thin Solid Films..

[9] Vereschaka AA (2018). Investigation of wear and diffusion processes on rake faces of carbide tool with Ti-TiN-(Ti,Al,Si)N composite nanostructured coating. Wear.

[10] Ranjan P, Hiremath SS (2019). Role of textured tool in improving machining performance: A review. J Manuf Process.

[11] Enomoto T, Sugihara T (2010). Improving anti-adhesive properties of cutting tool surfaces by nano-/micro-textures. CIRP Ann - Manuf Technol.

[12] Arulkirubakaran D, Senthilkumar V, Dinesh S (2017). Effect of textures on machining of Ti-6Al-4V alloy for coated and uncoated tools: A numerical comparison. Int J Adv Manuf Technol..

[13] Mishra SK, Ghosh S, Aravindan S (2018). Characterization and machining performance of laser-textured chevron shaped tools coated with AlTiN and AlCrN coatings. Surf. Coat. Technol..

[14] Charitha, R., Shrikantha, R. & Mervin, H. Performance improvement studies for cutting tools with perforated surface in turning of titanium alloy. International Conference on Research in Mechanical Engineering Sciences (RiMES 2017), MATEC Web of Conferences **144**, 03003 (2018).

[15] Sharma V, Pandey PM (2016). Comparative study of turning of 4340 hardened steel with hybrid textured self-lubricating cutting tool. Mater. Manuf. Processes..

[16] Yang Y, Su Y, Li L, Ning H, Zhao W (2015). Performance of cemented carbide tools with microgrooves in Ti-6Al-4V titanium alloy cutting. Int J Adv Manuf Technol..

[17] Ze W, Jianxin D, Yang C, Youqiang X, Jun Z (2012). Performance of the self-lubricating textured tools in dry cutting of Ti–6Al–4V. Int J Adv Manuf Technol..

[18] Chen ZM, Zhang JF, Feng PF, Wu ZJ (2013). A simulation study on the effect of microtextured tools during orthogonal cutting of titanium alloy Ti–6Al–4V. Appl Mech Mater..

[19] Yunsong L, Jianxin D, Guangyuan Y, Hongwei C, Jun Z (2013). Preparation of tungsten disulfide (WS2) soft-coated nano-textured self-lubricating tool and its cutting performance. Int J Adv Manuf Technol..

[20] Ma. J, Duong NH, Lei S (2015). 3D numerical investigation of the performance of microgroove textured cutting tool in dry machining of Ti–6Al–4V. Int J Adv Manuf Technol..

[21] Hao X, Chen X, Xiao S (2018). Cutting performance of carbide tools with hybrid texture. Int J Adv Manuf Technol.

[22] Kumar CS, Patel SK (2018). Effect of WEDM surface texturing on Al2O3/TiCN composite ceramic tools in dry cutting of hardened steel. Ceram. Int..

[23] Thakre A, Thakur A (2015). Study of behaviour of aluminium oxide nanoparticles suspended in SAE20W40 oil under extreme pressure lubrication. Ind. Lubr. Tribol..

[24] Kotia A, Ghosh SK (2015). Experimental analysis for rheological properties of aluminium oxide (Al2O3)/gear oil (SAE EP-90) nanolubricant used in HEMM. Ind. Lubr. Tribol..

[25] Kshatriya RB, Shaikh YI, Nazeruddin GM (2013). Synthesis of green metallic nanoparticles (nano particles) and applications. Orient J Chem..

[26] Oskam G (2006). Metal oxide nanoparticles: synthesis, characterization and application. J Sol-Gel Sci Technol..

[27] Goudarzi M, Ghanbari D, Salavati-Niasari M, Ahmadi A (2016). Synthesis and characterization of Al(oh)_3_, Al_2_o_3_ nanoparticles and polymeric nanocomposites. J Clust Sci..

[28] Shankar SS, Rai A, Ahmad A, Sastry M (2004). Rapid synthesis of Au, Ag and bimetallic cored Au core –Ag shell nanoparticle using neam (Azadirachta Indica) leaf broth. J. Colloid Interface Sci..

[29] Ahmed S, Saifullah S, Ahmad M, Swami BL, Ikram S (2016). Green synthesis of silver nanoparticles using Azadirachta indica aqueous leaf extract. J Radiat. Res. Appl. Sci..

[30] Benyounis KY, Olabi AG, Hashmi MSJ (2008). Multi-response optimization of CO_2_ laser-welding process of austenitic stainless steel. Opt. Laser. Technol..

